# Combined genetic approaches yield a 48% diagnostic rate in a large cohort of French hearing-impaired patients

**DOI:** 10.1038/s41598-017-16846-9

**Published:** 2017-12-01

**Authors:** D. Baux, C. Vaché, C. Blanchet, M. Willems, C. Baudoin, M. Moclyn, V. Faugère, R. Touraine, B. Isidor, D. Dupin-Deguine, M. Nizon, M. Vincent, S. Mercier, C. Calais, G. García-García, Z. Azher, L. Lambert, Y. Perdomo-Trujillo, F. Giuliano, M. Claustres, M. Koenig, M. Mondain, A. F. Roux

**Affiliations:** 1 Laboratoire de Génétique Moléculaire, CHU Montpellier, Montpellier, France; 2 Service ORL, CHU Montpellier, Montpellier, France; 3 Centre National de Référence Maladies Rares “Affections Sensorielles Génétiques”, CHU Montpellier, Montpellier, France; 4 Génétique Médicale, CHU Montpellier, Montpellier, France; 5 Service de Génétique, CHU-Hôpital Nord, Saint-Etienne, France; 6 Service de Génétique Médicale, CHU Nantes, Nantes, France; 7 Service de Génétique Médicale, CHU Toulouse, Toulouse, France; 8 Service d’ORL, Otoneurologie et ORL pédiatrique CHU Toulouse, Toulouse, France; 9 Service d’ORL, CHU Nantes, Nantes, France; 10 Laboratoire de Génétique de Maladies Rares (LGMR) EA7402, Université de Montpellier, Montpellier, France; 11 Génétique Médicale, Centre de Compétence des Surdités Génétiques, site constitutif du Centre de Référence des Anomalies du Développement et Syndromes Malformatifs de l’Est, CHRU Nancy, Nancy, France; 12 Service de Génétique Médicale, Centre de Référence pour les Affections Rares en Génétique Ophtalmologique (CARGO), Hôpital Civil, Strasbourg, France; 13 Service de Génétique Médicale, CHU Nice, Nice, France

## Abstract

Hearing loss is the most common sensory disorder and because of its high genetic heterogeneity, implementation of Massively Parallel Sequencing (MPS) in diagnostic laboratories is greatly improving the possibilities of offering optimal care to patients. We present the results of a two-year period of molecular diagnosis that included 207 French families referred for non-syndromic hearing loss. Our multi-step strategy involved (i) *DFNB1* locus analysis, (ii) MPS of 74 genes, and (iii) additional approaches including Copy Number Variations, *in silico* analyses, minigene studies coupled when appropriate with complete gene sequencing, and a specific assay for *STRC*. This comprehensive screening yielded an overall diagnostic rate of 48%, equally distributed between *DFNB1* (24%) and the other genes (24%). Pathogenic genotypes were identified in 19 different genes, with a high prevalence of *GJB2*, *STRC*, *MYO15A*, *OTOF*, *TMC1*, *MYO7A* and *USH2A*. Involvement of an Usher gene was reported in 16% of the genotyped cohort. Four *de novo* variants were identified. This study highlights the need to develop several molecular approaches for efficient molecular diagnosis of hearing loss, as this is crucial for genetic counselling, audiological rehabilitation and the detection of syndromic forms.

## Introduction

Hearing loss (HL) is the most common congenital sensory impairment in humans, and it affects approximately 1 in 600 newborns^1^. It is estimated that half of the cases have a genetic origin. HL can be non-syndromic (NSHL) and not associated with other clinical signs, or it can present as one of the symptoms in syndromic forms. In addition, some non-syndromic forms can evolve to syndromic forms later in life and are then defined as NSHL mimics^2^. The most common example is Usher syndrome (USH), which alters hearing and in some cases balance early in life, whereas it is only after the first decade that clinical signs of retinitis pigmentosa (RP) will affect the patient’s vision^3^.

Over 100 genes have been associated with NSHL and more still with syndromic HL^4^. Simultaneous screening of multiple genes is now possible with the advent of massively parallel sequencing (MPS). This approach offers the possibility of identifying the aetiology of the HL and thus providing proper genetic counselling to the families. Gene testing also impacts the clinical management of patients, as identifying the pathogenic alterations in syndromic genes of patients referred for NSHL will require a change or adaptation in care, as in the case of Usher syndrome^5^. Genetic findings can also indicate the need for additional clinical evaluation that may detect subtle syndromic features not necessarily related to the associated syndrome^6^. MPS improves diagnostic rates^7–9^. Although several approaches can be used, i.e. exome or gene panels, the latter have thus far shown the best compromise between the mutation detected rate and cost^2^. Studies of large cohorts of different origins have highlighted that the mutation detection rate depends on clinical characteristics and ethnicity^2,9^. Yet no data on the prevalence of HL genes and positive rates for diagnostic purposes are available for French patients. We have therefore tested a gene-panel approach over a two-year recruiting period for 207 families referred for NSHL from 14 centres distributed all over France. We present in this study an efficient decision-making process that identifies the genetic HL aetiology in 48% of patients.

## Results

We analysed a total of 207 index cases over a two-year period following the decision-making tree presented below.

### Decision-making tree

**Figure 1:**
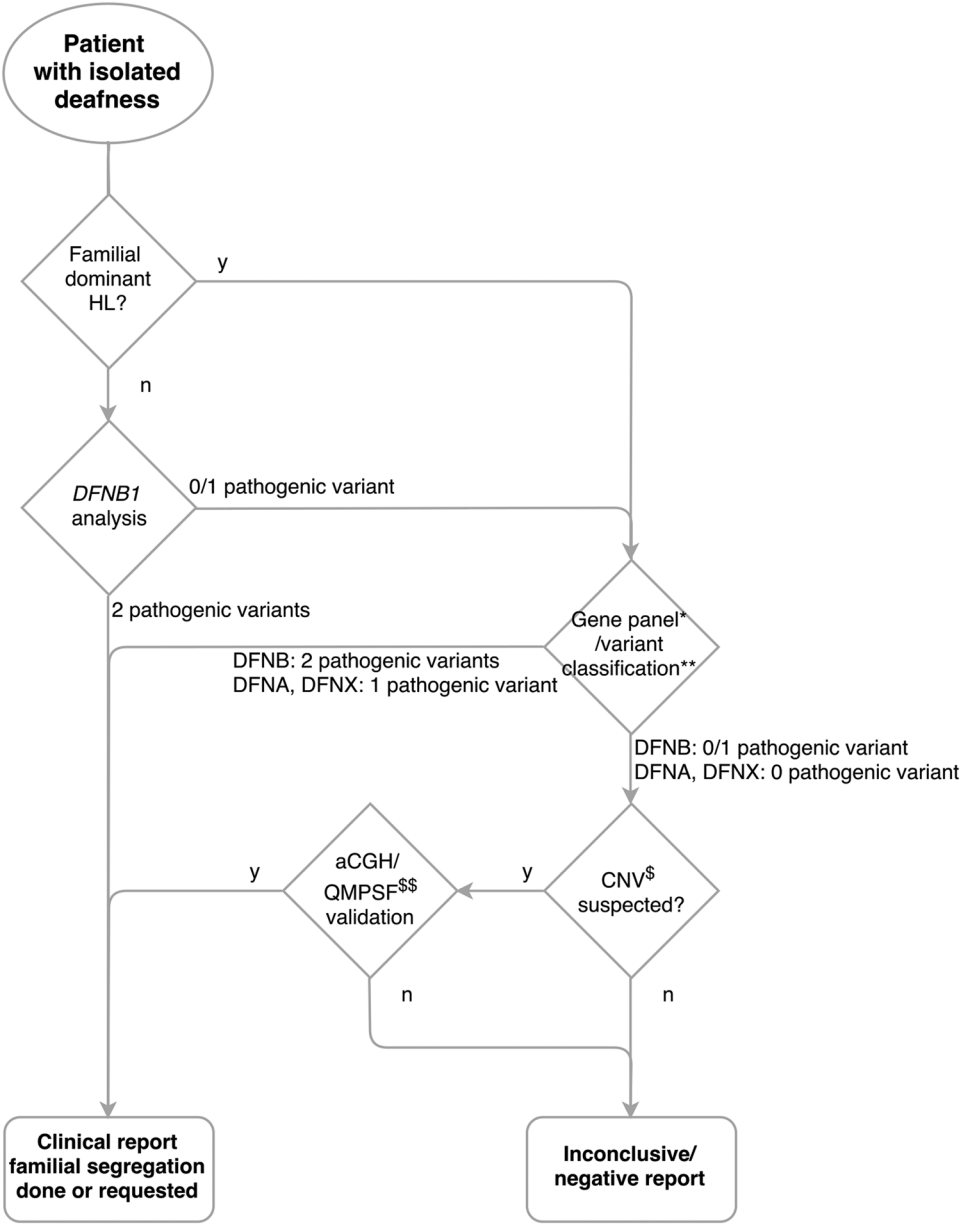
Decision-making tree for molecular diagnosis of isolated hearing loss. *74 HL genes, see Supplementary Table 1. **See Supplementary Figure 1. ^$^CNV: Copy Number Variation, deletions/duplications involving at least one exon and occurring in an isoform involved in hearing. ^$$^aCGH: array Comparative Genomic Hybridization; QMPSF: Quantitative Multiplex PCR of Short Fluorescent fragments. All SNVs and small indels are confirmed by Sanger sequencing or long-range PCRs followed by Sanger sequencing for *STRC* variants.

Analyses were performed following the tree presented on Fig. 1 (and Supplementary Table 1 and Supplementary Figure 1).

*DFNB1* analysis was performed for all cases with the exception of those patients presenting with a family history of dominant transmission. Although the *GJB2* and *GJB6* genes are included in the panel, *DFNB1* locus screening can be performed at reduced cost with a fast turn-around time. Patients with suspicion of Pendred/DFNB4 disease underwent the same strategy, even though SLC26A4 can be prioritized according to scan data.

### DFNB1 genotypes

Fifty-five patients were identified with pathogenic *DFNB1* variants (Supplementary Tables 2 and 3). Six patients carrying a single pathogenic variant were further analysed on the gene panel. Patient S1760, who was homozygous for the p.Met34Thr variant, was also included for further studies because the clinical data were not coherent with the identified genotype (profound HL and enlarged vestibular aqueduct: EVA).

The 49 *DFNB1* pathogenic genotypes included 20 different variants: although c.35delG represented 58% (57/98) of the pathogenic alleles, genetic heterogeneity was notable as most of the other variants were encountered only once or twice.

### Other NSHL genotypes

In 50 additional patients, pathogenic genotypes were identified in 18 different genes using MPS strategy (Supplementary Table 2). The data were categorized according to genotypes identified in truly non-syndromic genes and in those of the NSHL mimics (i.e. USH genes).

*STRC* pathogenic genotypes were identified in nine patients presenting with mild to moderate HL. Of these 18 mutant alleles, 12 consisted of large deletions of the gene. Although it was not possible to characterize the deletion breakpoints with our approach, we identified deletions of different sizes. The most frequent type encompassed the entire *STRC* gene and its 5′ sequence (c.(?_−78)_(*109_?)del), whereas the others maintained the 5′ end of the gene.

Patient S1537 was first identified as a heterozygous carrier of two alterations, a short deletion in intron 23 (c.4545+2_4545+6del) and a potential copy number variation (CNV) in exon 28. However, visual inspection of the aligned sequence reads using the Integrative Genomics Viewer (IGV) highlighted a reduced depth of coverage of *STRC*-exon 28 associated with an increased depth of ψ*STRC*-exon 28 reads, in comparison with control (Supplementary Figure 2). Therefore, this CNV was more likely to be a software alignment artefact. Sanger analysis with *STRC-*specific long-range PCR followed by nested PCR (LR/nested PCR) that was focused on exon 28 then identified the heterozygous substitution c.5125A > G in this patient S1537. This variant was already reported as pathogenic by Vona *et al*.^10^ and corresponds to an existing divergent nucleotide in the pseudogene sequence, explaining the misalignment of sequence reads observed with MPS technology.

**Figure 2:**
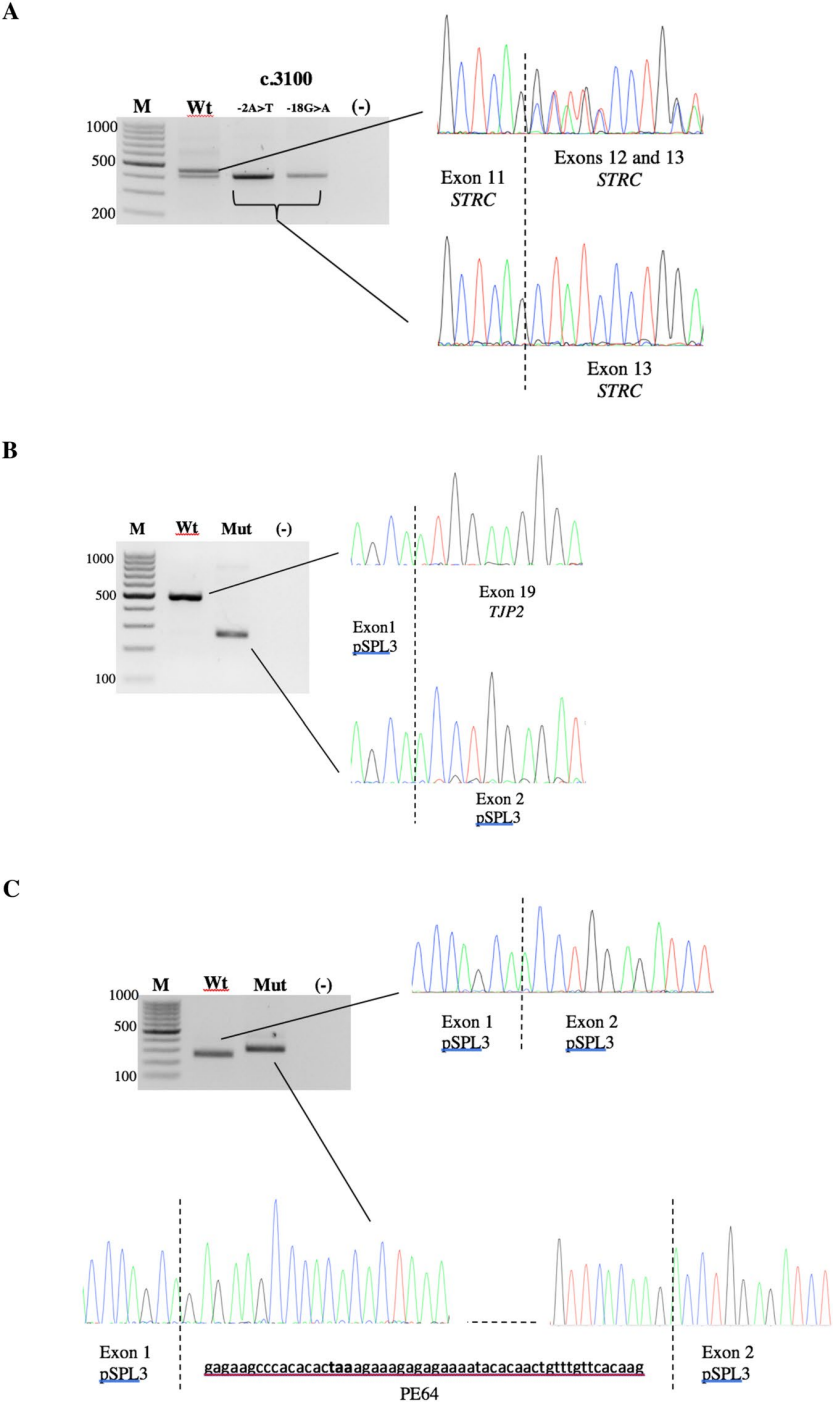
Minigene analysis to assess the impact of variants on splicing. Full-length gels showing: (**A**) *STRC* variants c.3100-2 A > T and c.3100-18 G > A leading to complete skipping of exon 12. The lower band observed for the wild-type construction may be due to an artefact of the minigene system or alternative splicing of exon 12; (**B**) *TJP2* c.2880G > A leading to complete skipping of exon 19; (**C**) *USH2A* variant c.14134-3169 A > G leading to the inclusion of a pseudoexon between native exons 63 and 64 (PE64); bold: termination codon.

IGV analyses were also conducted for S1511 and S1516, both of whom presented with moderate HL and carried a heterozygous deletion encompassing the *STRC* gene. In both cases, a substitution in intron 11 was identified by visual inspection (c.3100-2A > T for S1511 and c.3100-18G > A for S1516). Results of the LR/nested PCRs confirmed the *STRC* localization of the variants and minigene assays revealed in both cases complete skipping of exon 12, resulting in the loss of 13 amino acids (Fig. 2A).

*MYO15A*, *OTOF* and *TMC1* mutations were also identified in four, three and two patients, respectively. All identified mutations were associated with severe to profound HL.

All together more than a third (18/50) of the patients carrying a pathogenic genotype detected by MPS screening carried mutations in one of the four following genes: *STRC, MYO15A, OTOF* and *TMC1*.

Variants in rarely involved genes were also identified, such as the *de novo* c.2880G > A alteration in *TJP2* (patient S1324). Interestingly, this variant, located at the last nucleotide of exon 19, was not predicted to alter the protein sequence; the MaxEnt algorithm predicted a potential impact on splicing. Our minigene analysis confirmed that c.2880G > A indeed led to skipping of in-frame exon 19, which resulted in the deletion of amino acid residues 890–960 in the protein (Fig. 2B).

### Pathogenic genotypes in Usher genes

The 10 genes responsible for USH were included in our design. Sixteen of the 99 patients carried a pathogenic genotype in an Usher gene, the most frequent genes being *MYO7A* and *USH2A* in five and four patients, respectively. All patients, with the exception of two carrying mutations in *MYO7A*, were under 15 years and had been referred for NSHL, which is consistent with the onset of RP after the first decade.

Interestingly, MPS analysis on patient S1679 revealed a single *USH2A* heterozygous c.4645C > T nonsense variant. According to our diagnostic strategy, *USH2A*-whole-gene screening was then performed and detected a deep intronic substitution likely to affect splicing. This variation, c.14134-3169A > G in intron 64, predicted the creation of a strong donor splice site (MaxEntScan score: 7.64) potentially leading to a new pseudoexon (PE) activation. Minigene assay confirmed the pathogenic effect of this intronic variant on the splicing process and defined the size of the inserted pseudoexon PE64 (Fig. 2C). The latter, composed of 52 nucleotides, carried a premature termination codon (PTC) leading to a truncated protein if synthetized.

### Additional pathogenic variants

Ten of the 50 patients genotyped by MPS carried an additional pathogenic variant in another gene (Supplementary Table 2). Four corresponded to the *GJB2* heterozygotes initially screened at the *DFNB1* locus. Unsurprisingly, one of them was a c.35delG heterozygote and two other patients carried the p.(Met34Thr) alteration. This reflects the carrier frequencies of these two variants in the general population^11^. Two of the patients were *MYO15A* heterozygotes and another carried a deletion in *STRC*; again, these being two frequently involved genes in NSHL.

### 3D analysis

**Figure 3:**
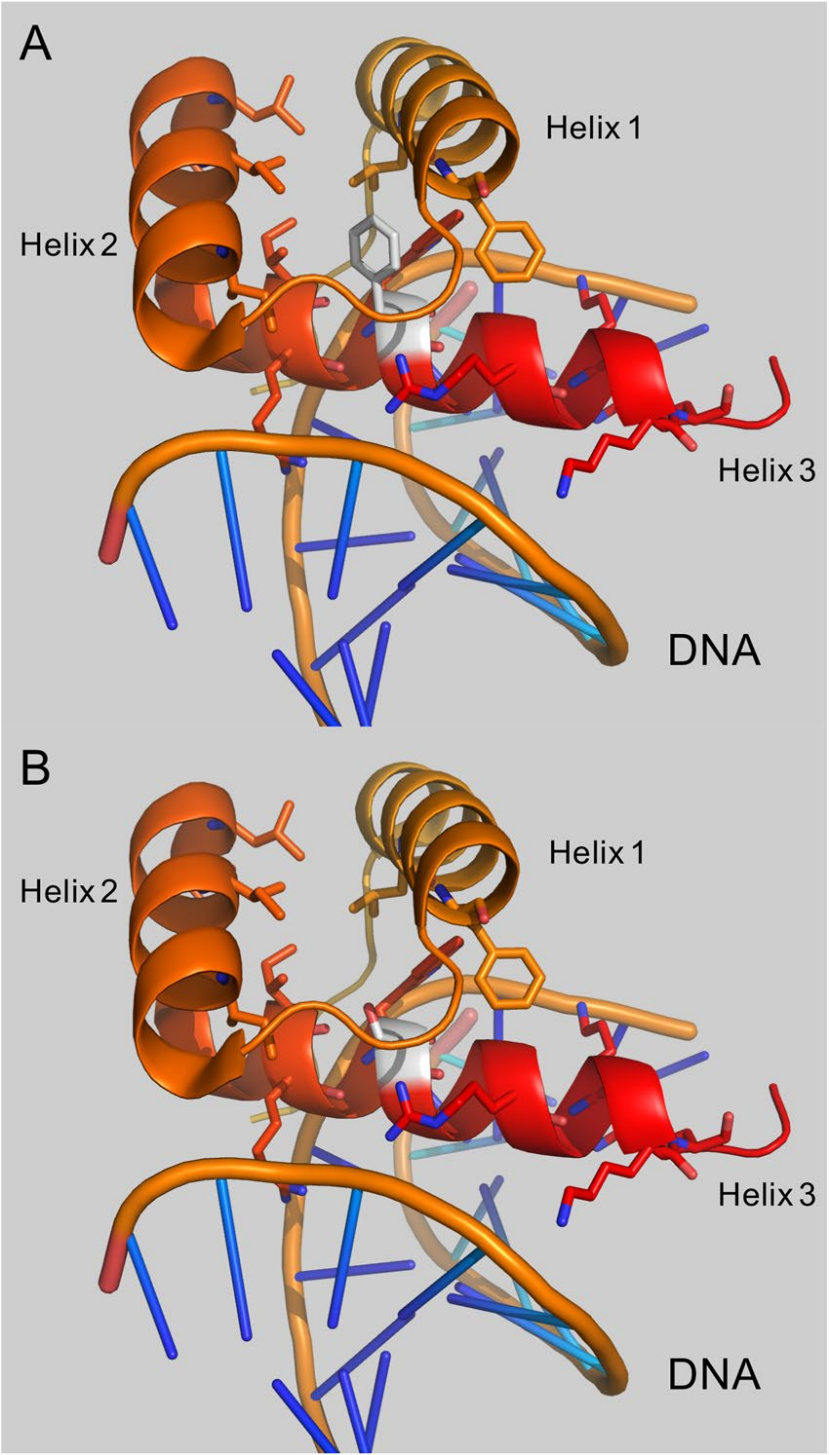
3D analysis of *POU4F3* p.(Phe322Ser) using homolog *Oct-1* PDB structure 1E3O. The homeodomain directly binds DNA through helix 3 and the positively charged amino acids Arg or Lys (red). The equivalent of Phe 322 (**A**, white), also located in helix 3, is involved in the hydrophobic core with helices 1 and 2 stabilizing the region. Introduction of a small polar amino acid (Ser, **B**, white) is likely to modify the hydrophobic core and the domain and might modify DNA binding properties of the protein.

In order to better characterize the missense variants, we performed a structural analysis using 3D modelling. This approach requires the availability of a crystallographic structure or a reliable model. For example, patient S1542 carried the *POU4F3* p.(Phe322Ser) variant and the structure showed a localization in helix 3 of the homeodomain in the model Oct-1 (*POU2F1*) protein. This helix directly binds DNA and the highly conserved Phe is part of the hydrophobic core involving helices 1 and 2 that stabilizes the helix-turn-helix conformation of the domain and allows the binding with DNA. Replacement of Phe with the small polar Ser would very likely destabilize the region and might impact DNA binding (Fig. 3). Familial segregation of this variant was further confirmed over a three-generation family that included six affected patients.

## Discussion

### Diagnostic rate

**Figure 4:**
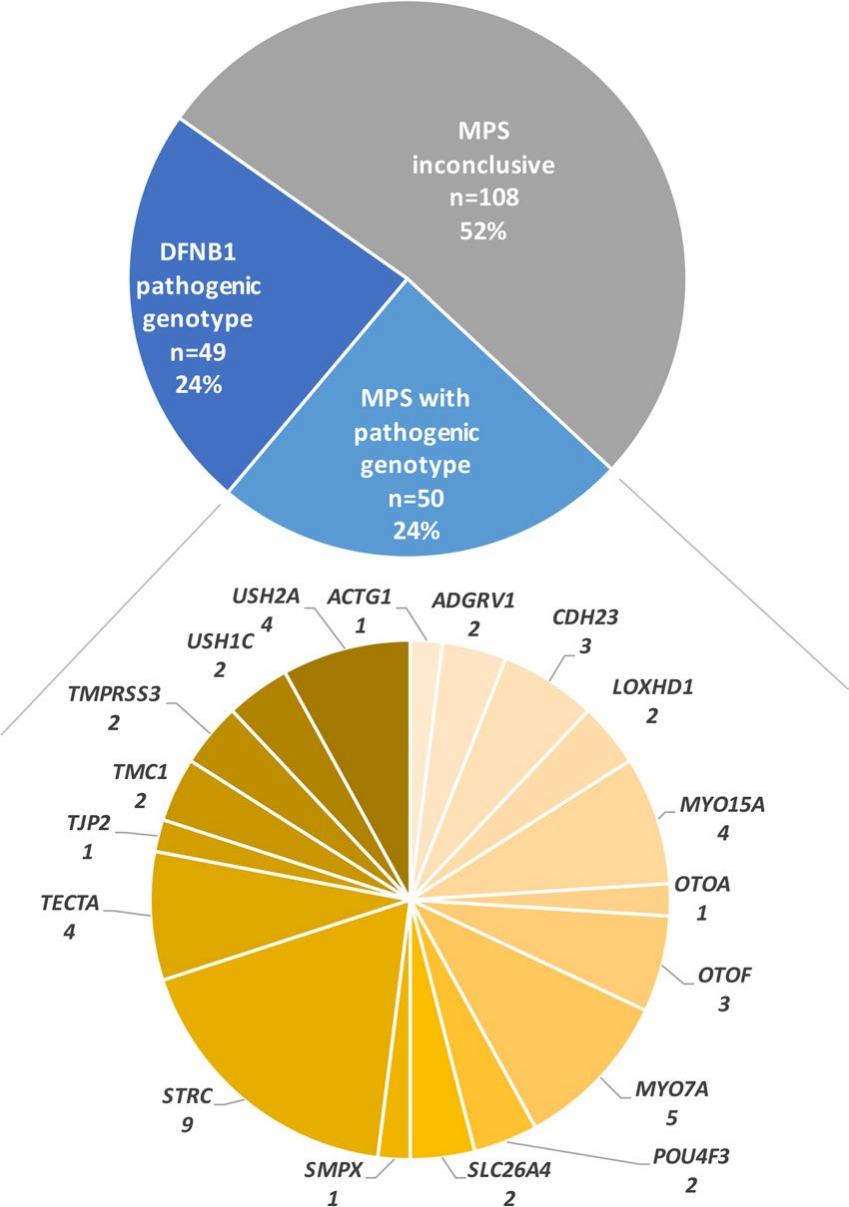
Diagnostic rates of the French NSHL cohort. After *DFNB1* screening, 158 patients underwent MPS and pathogenic genotypes could be defined for 50 of them, involving 18 different genes.

In order to establish an unbiased diagnostic rate, we included all patients referred for NSHL over a two-year period, without selecting the degree of HL or familial history. Following the described strategy, we were able to unambiguously genotype 48% of the patients (Fig. 4). Use of MPS drastically improved the diagnostic rate, by doubling the number of patients diagnosed with a confirmed genetic origin in our cohort. Nineteen different genes were causally implicated, but among them only five contributed to 71% of the cases: *GJB2* (*DFNB1* locus), *STRC*, *MYO7A*, *MYO15A* and *USH2A* (Fig. 4).

Two other studies testing large cohorts with different HL gene panels have recently reported diagnostic rates varying from 32^7^ to 56%^12^. However, in the latter, the high positive rate was explained by an enrichment of familial cases. Sloan-Heggen *et al*. provided data on the largest cohort (n = 1,119) with an overall rate of 39%. They showed that the positive rate varies greatly with degree of HL, inheritance of HL and ethnicity^2^. Comparing rates across different studies is difficult as many different parameters can interfere, from the composition of the cohort to the genes analysed.

One hundred and eight patients remained undiagnosed with no or a single identified pathogenic variant. For an unknown proportion of these patients, the HL is not of genetic origin. However, to improve the diagnostic rate of genetic cases, additional genes mimicking NSHL and involved in syndromic forms could be included in the panel^2^. Recently, Abou Tayoun *et al*. suggested that only genes with strong gene-disease association criteria should be included in diagnostics, and these authors provided a valuable core panel^13^. However, if genes with weak gene-disease association are not included, additional clues that a gene is indeed involved in HL might be missed. As an example, not including *TJP2* would have missed the diagnosis for patient S1324.

Using the gene panel approach for diagnostic purposes is still valuable as the quality remains superior to clinical exome or whole exome sequencing^14^. Therefore, a reasonable approach would be to include HL genes as well as candidates in a larger gene panel and to define a core diagnostic list following recommendations^13^. Diagnosis would be established quite confidently for genes included in the core list, whereas interpretation would be more cautious for the other genes.

### *STRC* analysis pitfalls

In accordance with other studies^2,7^, our results show that *STRC* was prone to large deletions and was clearly the second most frequent gene to be involved in NSHL. Tandem repeats are genomic features prone to instability and, as expected, large CNVs were the major mutational event for the *STRC* gene, with an estimated heterozygous deletion frequency of 1% to 1.6%^15^. In line with this high carrier rate, S1689 was identified as a heterozygous carrier of a *STRC* deletion (Supplementary Table 3). *STRC* was most likely not involved as this patient presented with profound hearing loss.

Overall, because of the high sequence homology between *STRC* and ψ*STRC*, specific approaches need to be developed to detect the large rearrangements as well as the point mutations that can be masked by the ψ*STRC* sequences.

### *De novo* occurrence of variants

*De novo* variants were observed in several cases involving both DFNA or DFNB/USH genes. The *ACTG1* variant (c.434C > T, p.(Ser145Phe)) arose *de novo* in S1572. This variant was confirmed to be absent in both parent samples. Mutations in *ACTG1* can be associated with either Baraister-Winter syndrome^16^ or NSHL^17^. In the present case, the young patient presented only NSHL with normal facial appearance, although MRI was not performed. At birth, otoacoustic emissions (OAEs) were recordable and hearing loss was moderate but evolved into profound within three years. *De novo* variants of this gene have already been described in several Baraister-Winter cases^18^.

A *de novo TJP2* variant (c.2880G > A) was also identified in patient S1324. Minigene analysis demonstrated an effect on the splicing process (Fig. 2B), and the implication of *TJP2* in NSHL remains questionable. The progression of HL was noted over 15 years, altering mainly high frequencies. The *de novo* occurrence of the variant together with the alteration in *TJP2* splicing reinforces the hypothesis that this gene is indeed involved in NSHL.

Familial segregation performed for S1759 revealed that neither parent was a carrier of the *TECTA* p.(Pro2079Leu) variant, suggesting a *de novo* occurrence. The residue Pro was perfectly conserved among 89 orthologs (UCSC MultiZ alignment, not shown) and was located within a highly-conserved region of the protein. The patient presented with a moderate congenital HL with a flat configuration; as he was under 2 years of age, an evolution in HL cannot be excluded.

Last, patient S701 was found to carry two alterations in the *CDH23* gene. But as the c.6254-2A > G variant arose *de novo*, it was not possible to determine whether the two alterations are *in trans*. At this date, a question remains about a possible Usher syndrome.

All patients were initially sent for isolated, sporadic NSHL and these findings pinpoint the importance of systematic segregation analyses as identification of *de novo* variants in dominant forms of NSHL will have a direct impact on transmission risk and further genetic counselling.

### Correlating genotypes with phenotypes for NSHL

This study confirms that *TMPRSS3* is involved in progressive HL. The patient audiograms had a characteristic ski-slope configuration as previously shown^19^. The *MYO15A* and *TMC1* mutations are mainly associated with severe to profound HL, whereas *STRC* alterations lead to mild to moderate hearing loss. All patients carrying *OTOF* mutations presented with profound HL. Most of the variants led to PTC and loss of functional protein. Recent data suggest that some missense variants can act as hypomorphic alleles and can be associated with moderate HL^20^. It is likely that in the future we will also extend the phenotypic spectrum associated with *OTOF* variants.

### Questions linked to the identification of pathogenic genotypes in genes mimicking NSHL

We identified a USH pathogenic genotype in 16% of the patients. All patients were initially referred for NSHL. After genotyping, two groups should be distinguished based on age, i.e. those with NSHL mimics and those with true NSHL.

*CDH23, MYO7A* and *USH1C* have long been known to be involved in USH as well as non-syndromic hearing loss^21–23^. Therefore, it is possible that not all patients with mutations in these genes will develop a typical Usher syndrome. For example, patient S1692 is 27 years old and the electroretinogram (ERG) performed after the molecular results was found to be subnormal. Consequently, this patient is not expected to develop a typical Usher syndrome and signs of RP may remain very subtle throughout his lifetime. Similarly, S1741, S1763 and S1799 underwent further clinical examination after the molecular results and it is suspected that all will develop Usher syndrome. The awareness of future development of RP will have an impact on audiological rehabilitation and the genetic counselling for the families. In several cases, prenatal diagnosis could thus be offered for the next pregnancies. Last, patients carrying *USH1C* (S1536, S1707), *USH2A* (S1682, S1679, S1752 and S1786) and *ADGRV1* variants (S1338 and S1601) were referred to ophthalmology clinics in order to establish their visual function.

### Additional tools are required for optimized diagnostic service

In this study, we provide a rather comprehensive approach through the development of interpretation tools. Indeed, when possible, 3D analysis can provide additional clues to classify a variant, which is shown with the example of the *POU4F3* p.(Phe322Ser) variant (Fig. 3).

An additional step for assessing missense pathogenicity would be to perform *in vitro* functional tests, whose feasibility has been demonstrated for genes such as *SLC26A4* ^24^.

We previously showed the usefulness of integrating minigene analysis for detailed study of the consequences of predicted splicing alterations^25,26^. Again, four variants identified in this cohort could be confirmed as affecting splicing by minigene analysis (Fig. 2).

In addition, because several *USH2A* deep intronic mutations have already been identified^26,27^, we recommend performing either transcript analysis or whole *USH2A* gene sequencing for patients carrying a single *USH2A* pathogenic variant. This allowed the identification of a novel deep intronic mutation in intron 64, leading to a new PE insertion. This mutation has since been identified in another patient presenting with Usher type 2 (unpublished results). All together, this is the fifth deep intronic mutation resulting in PE insertion identified in *USH2A*, thus confirming the particularly high rate of this type of alteration in the most frequent USH gene.

In conclusion, this study based on the genetic screening of 207 patients referred for NSHL shows that MPS clearly improves the molecular diagnosis of NSHL in the French population, as already shown in other populations. However, this diagnostic rate of 48% was achieved using MPS plus other approaches aimed at characterizing the impact of variants or confirming their actual existence (*in silico* and minigenes studies, CNVs analysis), or identifying them in the case of *STRC* variants and *USH2A* deep intronic variants (customized whole gene screening). In addition, a high proportion of our cohort presented with pathogenic genotypes in USH genes, which should thus definitely be included in NSHL screening as their involvement immediately modifies the medical care for both patients and their families.

## Methods

### Patient recruitment

All patients included in this study were referred from medical genetics or ENT departments for apparent NSHL. They or their parents responded to a clinical questionnaire for family history and audiograms were performed to evaluate their degree of HL.

Informed consent to genetic testing was obtained from adult probands or parents in the case of minors after explanation of the nature and its possible implications for the patient and his family. This study was performed in accordance with the French law on bioethics: ‘loi de bioéthique’, revised 7 July 2011, number 2011-814. The experimental protocol is approved by the Montpellier University Hospital (CHU Montpellier) as part of the molecular diagnostic activity. The authorization number given by the Agence Régionale de la Santé (ARS) is LR/2013-N°190. Nominative licensing in the name of Dr. Roux was delivered by Agence de la Biomédecine.

### Hearing loss assessment

Audiometric data were collected from the various departments and obtained using calibrated audiometers according to the International Organization for Standardization ISO^28^. In adults and children of at least 6 years old, bilateral air and bone conduction hearing thresholds (dB HL) were obtained for 0.25, 0.5, 1, 2, 4 and 8 kHz frequencies. For young children unable to undergo bilateral evaluation, open-field air and bone conduction thresholds were assessed using age-appropriate procedures (behavioural observation audiometry, visual reinforced audiometry or play audiometry). An electrophysiological assessment completed the audiological evaluation if necessary (i.e. auditory brainstem response thresholds, auditory steady state response, acoustic otoemission). Data from universal newborn screening were collected for the youngest patients.

Hearing loss degree was analysed according to the International Bureau for Audiophonology audiometric classification (https://www.biap.org/en/recommandations/recommendations/tc-02-classification/213-rec-02-1-en-audiometric-classification-of-hearing-impairments/file), whereas other phenotype features were analysed according to the recommendations for the description of non-syndromic hearing loss^29^. Sensorineural hearing loss was found in all cases.

### *DFNB1* locus screening

The screening was performed as recommended by the EMQN *DFNB1* guidelines^30^ and included Sanger sequencing of *GJB2* exons 1 and 2 and their flanking intronic regions and multiplex PCR as designed by del Castillo *et al*.^31^ to detect the two large deletions encompassing part of the *GJB6* gene, del(GJB6-D13S1830) and del(GJB6-D13S1854).

Sequences were compared with the reference sequence using the Seqscape 2.5 software.

### Gene-panel sequencing and bioinformatics

All patients underwent MPS gene-panel testing if *DFNB1* locus screening showed no evidence of a pathogenic genotype (Fig. 1). Two panels were used. The first one included 65 genes and was designed with Illumina® Nextera Rapid Capture Custom Enrichment technology. An updated design of 74 DFN genes was performed and used for most of the patients (Supplementary Table 1) with NimbleGen SeqCap EZ Choice technology. All patients negative for the first panel were analysed with the updated 74 NSHL gene panel. The pros and cons of both enrichment technologies are detailed in^32^. Both designs were targeting the exons referenced in RefSeq or Ensembl (coding and non-coding) with 50 bp surroundings. Sequencing was performed on either an Illumina MiSeq instrument (using version 2 chemistry) or an Illumina MiniSeq system. As the two instruments have a similar output, the number of samples per run was the same (12 samples). The secondary analysis (mainly alignment and variant calling) was performed using the commercial software MiSeqReporter (v2.5) for the MiSeq runs and LocalRunManager (v1.3.1) for the MiniSeq runs, these workflows being very similar. Variant Calling Files (VCFs) were automatically included in our in-house database system (USHVaM2), which also handled variant annotation. In addition, when the pathogenic genotype was unclear, the samples were re-analysed using an in-house pipeline (Nenufaar, https://github.com/mobidic/nenufaar), which performed the secondary analysis and annotation of the variants. In particular, Nenufaar uses more recent software than the commercial pipelines and is better at retrieving small indels. Last, variants of interest were all confirmed by Sanger sequencing.

When *STRC* variants were identified, confirmation was performed by nested PCR. Briefly, *STRC* nested PCRs consist of a first long-range amplification (LR-PCR) excluding the ψ*STRC* pseudogene followed by nested amplifications targeting exons of interest and exon 20 to confirm pseudogene exclusion. The GoTaqLong PCR Promega kit (Promega) LR-PCR was used for LR-PCR according to the manufacturer’s recommendations and with primers described lesewhere^33^. LR-PCR products were diluted 1:1000 and then used for the nested PCRs. Primer sequences and reaction conditions for nested PCR are available on request.

### Whole *USH2A* sequencing

Whole *USH2A* sequencing was performed for S1679 as already described by Liquori *et al*.^26^. Following the defined thresholds, a single variant susceptible to alter splicing was tested by minigene analysis.

### Detection of copy number variants (CNVs)

In order to highlight the potential CNVs, which are not retrieved by the described pipelines, we used an in-house spreadsheet that computes the inter-sample normalized depth of coverage per exon in a given run. Potential CNVs were validated by array CGH using a Sure Print G3 CGH personal 4*180K custom design for 26 HL genes labelled with the SureTag Complete labelling kit. The array was analysed on an Agilent DNA microarray scanner C. For the remaining genes, quantitative multiplex PCR of short fluorescent fragments (QMPSF) designed with specific labelled primers was used when necessary.

In particular, suspected large deletions of *STRC* were confirmed by QMPSF using four sets of primers targeting specific *STRC* sequences (5′ sequence: forward: 5′-TAGCTGGGATTACAGGTGC-3′, reverse: FAM5′-CATTCACTACCGGGCGTAG; 3′ sequence: forward: 5′-GTTGCACCAGCTCCACCTAAG-3′, reverse: FAM5′-TGAGATCCTAAGGGATTAGGAC; Intron 16: forward: 5′-AGTGTTTGGTCCATTGTAAAGTC-3′, reverse: FAM5′-CCATTGTTCTTCTAATGTGGGTG; Intron 22: forward: 5′-TGGGTTCTACATGTGCTCTTCC-3′, reverse: FAM5′-TACAGAATTCTAGAACTACAAGAGG-3′) and one additional set screening exon 18 of the *USH2A* gene, as a control (forward: 5′-AAGTAACCCCTTTGTCTGATGAGT-3′, reverse: FAM5′-AAGACTCTGAACTCATACTTGGTG-3′).

For potential short *STRC* CNVs, binary alignment map (BAM) files were loaded into the integrative genomics viewer (IGV) software (v2.3.55)^34^ in order to globally inspect the aligned sequence reads between the *STRC* gene and the ψ*STRC* pseudogene.

Point mutations were validated with a long-range PCR assay specific to the *STRC* gene (specificity of the amplification was ascertained by the absence of the divergent c.4057C > T base pair) followed by nested PCRs.

### Variant classification

Variants were classified using a fully described method^35^ (Supplementary Figure 1) that includes six classes from neutral to pathogenic and four classes of variants of unknown clinical significance. Variants predicting the inclusion of a PTC or located at canonical splicing positions (−2,−1, +1, +2 positions around exons) of isoforms described in pathology were considered *a priori* pathogenic. The main characteristics for missense classification were the familial segregation of the variant, its frequency in public databases, evolutionary conservation throughout orthologs and paralogs (domain conservation) and, when available, the impact on the 3D structure.

3D analysis of NP_002691.1: p.(Phe322Ser) was performed using human Oct-1 homeodomain encoded by *POU2F1* (Protein Data Bank ID: 1E3O) binding DNA as the model. 1E3O shares 56% amino acids identity with pou4f3 through residues 182–332 and 66% (12/18) for residues surrounding pou4f3 position 322 in helix 3 (residues 141–158). The pictures were built using the PyMOL software (The PyMOL Molecular Graphics System, Version 1.8 Schrödinger, LLC).

The potential impact on splicing for rare missense, isosemantic and intronic variants was also assessed using a local implementation of the MaxEnt algorithm^36^ and, when available, using dbscSNV results^37^. Minigene analysis was performed to experimentally verify the impact of selected variants on the splicing process, as described alsewhere^38^.

### Data Availability

All DNA variants identified during the course of this study have been deposited into the public LOVD3 shared genetic database: https://databases.lovd.nl/shared/ or the public LOVD2 “Retinal and hearing impairment genetic mutation database” (https://grenada.lumc.nl/LOVD2/Usher_montpellier/).

## Electronic supplementary material

Supplementary Information

Supplementary Table 2
