# Supplementary material for: Combined genetic approaches yield a 48% diagnostic rate in a large cohort of French hearing-impaired patients
Source: Sci Rep. 2017 Dec 1;7:16783. doi: 10.1038/s41598-017-16846-9 (PMC5711943; doi:10.1038/s41598-017-16846-9)
Supplement: Supplementary file 1 — Supplementary Information [file 41598_2017_16846_MOESM1_ESM.pdf]

Combined genetic approaches yield a 48% diagnostic rate in a large cohort of French hearing-impaired patients

Baux D[$] (1), Vaché C[$] (1), Blanchet C (2, 3), Willems M (4), Baudoin C (1), Moclyn M (1), Faugère V (1), Touraine R (5), Isidor B (6) , Dupin-Deguine D (7, 8), Nizon M (6), Vincent M (6), Mercier S (6), Calais C (9), Garcia-Garcia G (10), Azher Z (10), Lambert L (11), Perdomo-Trujillo Y (12), Giuliano F (13), Claustres M (1, 10), Koenig M (1, 10), Mondain M (2, 3), Roux AF (1, 10) *

[$] The first two authors contributed equally to this work

[1] Laboratoire de Génétique Moléculaire, CHRU Montpellier, Montpellier, France

[2] Service ORL, CHRU Montpellier, Montpellier, France

[3] Centre National de Référence Maladies Rares "Affections Sensorielles génétiques », CHU Montpellier, Montpellier, France

[4] Génétique Médicale, CHRU Montpellier, Montpellier, France

[5] Service de Génétique, CHU-Hôpital Nord, Saint-Etienne, France

[6] Service de Génétique Médicale, CHU Nantes, Nantes, France

[7] Service de Génétique Médicale, CHU Toulouse, Toulouse, France

[8] Service d'ORL, Otoneurologie et ORL pédiatrique CHU Toulouse, Toulouse, France

[9] Service d'ORL, CHU Nantes, Nantes, France

[10] Laboratoire de Génétique de Maladies Rares (LGMR) EA7402, Université de Montpellier, Montpellier, France

[11] Génétique Médicale, Centre de Compétence des Surdités Génétiques, site constitutif du Centre de Référence des Anomalies du Développement et Syndromes Malformatifs de l'Est, CHRU Nancy, Nancy, France

[12] Service de Génétique Médicale, Centre de Référence pour les Affections Rares en Génétique Ophtalmologique (CARGO), Hôpital Civil, Strasbourg, France

[13] Service de Génétique Médicale, CHU Nice, Nice, France

* Corresponding author: Anne-Françoise Roux, Laboratoire de Génétique Moléculaire, CHRU Montpellier, IURC, 641 Avenue du Doyen Gaston Giraud, F-34093 Montpellier cedex 5, France, anne-francoise.roux@inserm.fr

Supplementary Information

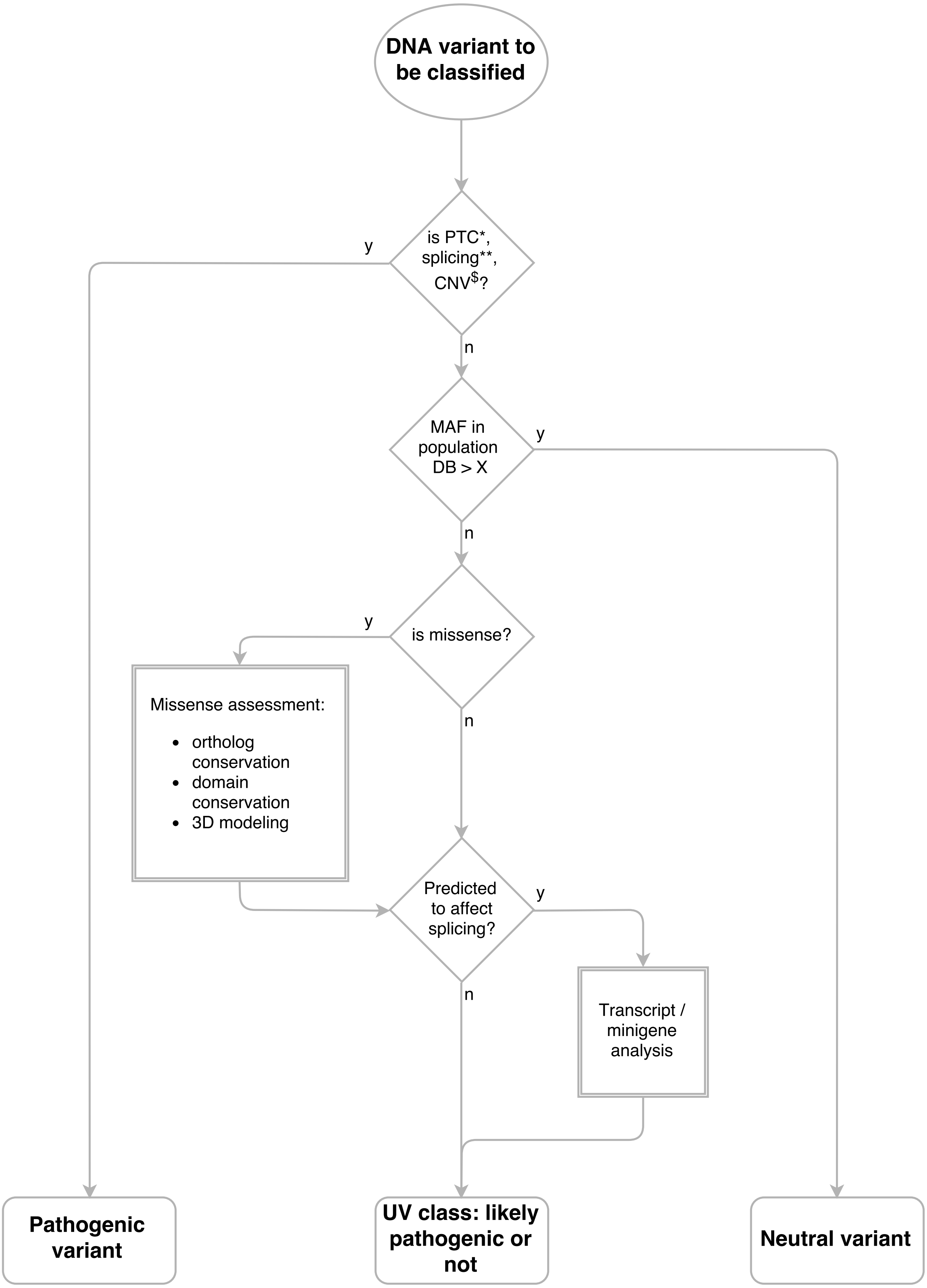

Supplementary Figure 1: Decision tree for variant classification
*: PTC: Premature Termination Codon
**: variant occurring at -2,-1,+1,+2 positions around exons that disrupt natural splice sites
$: CNV: Copy Number Variation, deletions/duplications involving at least one exon and occuring in an isoform involved in hearing
X: 0.01 for DFNB, 0.005 for DFNX, DFNA

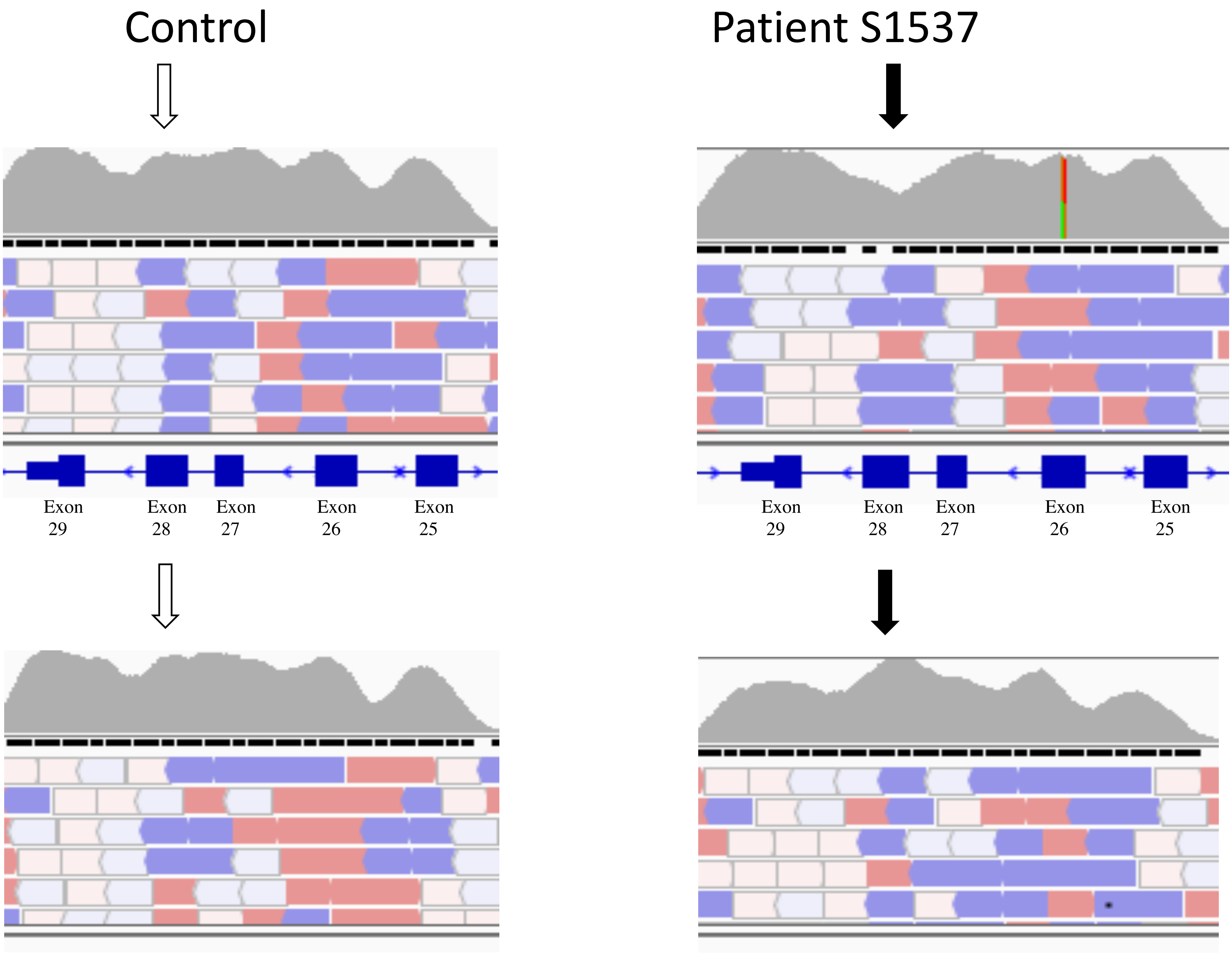

Supplementary Figure 2: IGV view of *STRC* and ψ*STRC* sequences from control and patient S1537. Differences, indicated by arrows, are visible for exons 28 suggesting a software alignment artefact. IGV, Integrative Genomics Viewer.

	HGNC Gene name	RefSeq accession number			
**DFN**	*ACTG1**	NG_011433.1		*LRTOMT**	NG_021423.1
	*ADGRV1**	NG_007083.1		*MARVELD2**	NG_017201.1
	ADCY1	NG_034198.1		*MIR96**	NG_023441.1
	*CABP2**	NG_032982.1		*MSRB3**	NG_023385.1
	*CCDC50**	NG_008994.1		*MYH14**	NG_011884.1
	*CDH23**	NG_008835.1		*MYH9**	NG_011645.1
	*CEACAM16**	NG_032692.2		*MYO7A**	NG_009086.1
	*CIB2**	NG_033006.1		*MYO3A**	NG_011635.1
	*CLDN14**	NG_031965.1		*MYO6**	NG_009934.1
	CLIC5	NG_011777.1		*MYO15A**	NG_011634.1
	*CLRN1**	NG_009168.1		*OTOA**	NG_012973.1
	*COCH**	NG_008211.2		*OTOF**	NG_033191.1
	*CRYM**	NG_011610.1		*OTOG*	NG_033008.1
	*DFNA5**	NG_011593.1		*OTOGL*	NG_009937.1
	*DFNB31**	NG_016700.1		*PCDH15**	NG_009191.1
	*DFNB59**	NG_029459.1		*POU3F4**	NG_009936.2
	*DIABLO**	NG_011594.1		*POU4F3**	NG_011885.1
	*DIAPH1**	NG_032693.1		*PRPS1**	NG_008407.1
	*DIAPH3**	NG_012186.1		*PTPRQ**	NG_034052.1
	EPS8	NG_041808.1		*RDX**	NG_023044.1
	*ESRRB**	NG_012278.1		*SERPINB6**	NG_027692.1
	*EYA4**	NG_011596.1		*SLC17A8**	NG_021175.1
	FOXI1	NG_012068.1		*SLC26A4**	NG_008489.1
	*GIPC3**	NG_031943.1		*SLC26A5**	NG_023055.1
	*GJB2**	NG_008309.1		*SMPX**	NG_031916.1
	*GJB3**	NG_008358.1		*STRC**	NG_011636.1
	*GJB6**	NG_008323.1		*TBC1D24*	NG_028170.1
	*GPSM2**	NG_028108.1		*TECTA**	NG_016342.1
	*GRHL2**	NG_011971.1		*TJP2**	NG_011633.1
	*GRXCR1**	NG_027718.1		*TMC1**	NG_008213.1
	GRXCR2	NG_034161.1		*TMIE**	NG_011628.1
	*HGF**	NG_016274.1		*TMPRSS3**	NG_011629.1
	*ILDR1**	NG_031870.1		*TPRN**	NG_027801.1
	KCNJ10	NG_016411.1		*USH1C**	NG_011883.1
	*KCNQ4**	NG_008139.1		*USH1G**	NG_007882.1
	*LHFPL5**	NG_012184.1		*USH2A**	NG_009497.1
	*LOXHD1**	NG_016646.1		*WFS1**	NG_011700.1

Supplementary Table 1 : list of genes analysed with the 74 DFN genes panel.
*indicates the genes already included in the previous panel.

	clinical information						Allele 1				
**Patient #**	**transmission**	**degree**	**onset**	**evolution**	**other symptom**	**2016 Age**	**Gene/locus**	**Transcript**	**chromosomal location hg38**	variant	**rs ID**
S1499	Sporadic	Profound	Congenital	_	_	4	*OTOF*	NM_194248.2	chr2:g.26477210G>A	c.2485C>T - p.(Gln829*)	rs80356593
S1510	Sporadic	Profound	Congenital	_	EVA	6	*SLC26A4*	NM_000441.1	chr7:g.107683538G>A	c.1001+1G>A - IVS8+1G>A	rs80338849
S1557	Sporadic	Severe-profound	Congenital	_	Normal CT scan	2	*MYO7A*	NM_000260.3	chr11:g.77157389dup	c.846dupC - p.(Met283Hisfs*3) + c.833_841del - p.(Tyr278_Tyr280del)	
S1565	Sporadic	Moderate	Before 4	Stable	Normal CT scan	31	*TMPRSS3*	NM_024022.2	chr21:g.42388532C>T	c.323-6G>A - IVS4-6G>A	rs374793617
S1660	Sporadic	Moderate	Before 7	_	_	6	*GJB2*	NM_004004.5	chr13:g.20189481A>G	c.101T>C - p.(Met34Thr)	rs35887622
S1664	Sporadic	Profound			Vestibular dysplasia; agenesie du canl auditif	7	*GJB2*	NM_004004.5	chr13:g.20189547del	c.35delG - p.(Gly12Valfs*2)	rs1801002
S1663	_	Moderate	Before 10	_	_	14	*MYO7A*	NM_000260.3	chr11:g.77205598C>T	c.5617C>T - p.(Arg1873Trp)	rs397516321
S1685-p	Sporadic	Profound	Congenital	Stable	_	27	*MYO7A*	NM_000260.3	chr11:g.77197568T>C	c.4411T>C - p.(Ser1471Pro)	rs397516310
S1687	Sporadic	Mild	Before 3	Stable	Normal CT scan	6	*OTOG*	NM_001277269.1	chr11:g.17635188G>A	c.7729+1G>A - IVS45+1G>A	rs548496846
S1689	Sporadic	Profound	Congenital	_	Normal CT scan	2	*STRC*	NM_153700.2	chr15:g.(?_43599563)_(436188	c.(?_-78)_(*109_?)del - p.? - (E1-3UTRdel)	
S1690	AR	Before 10	Severe	Evolutive	Normal CT scan	25	*PTPRQ*	NM_001145026.1	chr12:g.80649671T>C	c.6024+2T>C - IVS37+2T>C	
S1698	Sporadic	Severe	_	_	Normal CT scan	8	*OTOG*	NM_001277269.1	chr11:g.17611321del	c.6057delC - p.(Thr2020Glnfs*11)	
S1719	Sporadic	Severe	Congenital	_	_	9	*HGF*	NM_000601.4	chr7:g.81707309G>A	c.1597C>T - p.(Arg533*)	rs527951814
S1739	AD	Moderate	Before 3	_	Normal CT scan	7	*SLC26A4*	NM_000441.1	chr7:g.107710135A>G	c.2171A>G - p.(Asp724Gly)	
S1744	AD	Moderate-severe	Before 3	Stable	Mondini	15	*USH2A*	NM_206933.2	chr1:g.215817143C>A	c.9424G>T - p.(Gly3142*)	rs397518048
S1757	AD	Moderate	After 25	Progressive	en U	36	*MYO3A*	NM_017433.4	chr10:g.26211952C>T	c.4840C>T - p.(Gln1614*)	rs146106052
S1785	AD	_	_	_	Normal MRI	6	*WFS1*	NM_006005.3	chr4:g.6301894G>A	c.2099G>A - p.(Trp700*)	rs372114182
S1804	Sporadic	Moderate-profound	17 years	_	Normal CT scan; normal MRI	18	*GJB6*	NM_006783.4	chr13:g.20222792dup	c.689dupA - p.(Asn230Lysfs*11)	rs398124237

p: partner of S1685

Supplementary Table 3:
18 incomplete pathogenic genotypes and associated phenotypic information
